# Redefining Non-Motor Symptoms in Parkinson’s Disease

**DOI:** 10.3390/jpm15050172

**Published:** 2025-04-26

**Authors:** Laura Peña-Zelayeta, Karen M. Delgado-Minjares, Marcos M. Villegas-Rojas, Karen León-Arcia, Alberto Santiago-Balmaseda, Jesús Andrade-Guerrero, Isaac Pérez-Segura, Emmanuel Ortega-Robles, Luis O. Soto-Rojas, Oscar Arias-Carrión

**Affiliations:** 1 Unidad de Trastornos del Movimiento y Sueño, Hospital General Dr. Manuel Gea González, Ciudad de México 14080, Mexico; ladanyzel@gmail.com (L.P.-Z.); emmanuel.ortega@salud.gob.mx (E.O.-R.); 2 Laboratorio de Patogénesis Molecular, Laboratorio 4, Edificio A4, Carrera Médico Cirujano, Facultad de Estudios Superiores Iztacala, Universidad Nacional Autónoma de México, Tlalnepantla 54090, Mexico; km95.delgado@gmail.com (K.M.D.-M.); marcusvillegas3@gmail.com (M.M.V.-R.); klarci@comunidad.unam.mx (K.L.-A.); albertosantiago@comunidad.unam.mx (A.S.-B.); jesusandrade1007@gmail.com (J.A.-G.); dr.isaacps91@gmail.com (I.P.-S.); 3 Departamento de Fisiología, Biofísica y Neurociencias, Centro de Investigación y de Estudios Avanzados del Instituto Politécnico Nacional, Ciudad de México 07360, Mexico; 4 Sección de Estudios de Posgrado e Investigación, Escuela Superior de Medicina, Instituto Politécnico Nacional, Ciudad de México 11340, Mexico; 5 Departamento de Neurobiología del Desarrollo y Neurofisiología, Instituto de Neurobiología, Universidad Nacional Autónoma de México, Querétaro 76230, Mexico; 6 Posgrado en Ciencias Biológicas, Unidad de Posgrado, Edificio A, 1° Piso, Circuito de Posgrados, Ciudad Universitaria, Coyoacán 04510, Mexico; 7 Experimental Neurology, Instituto Nacional de Rehabilitación Luis Guillermo Ibarra Ibarra, Mexico City 14389, Mexico

**Keywords:** Parkinson’s disease, non-motor symptoms, sensory dysfunction, sleep-related disorders, neuropsychiatric disorders, autonomic dysfunction, REM sleep behavior disorder, pain, cognitive impairment, biomarkers

## Abstract

Parkinson’s disease involves widespread neurodegeneration that extends far beyond the basal ganglia, giving rise to a diverse range of non-motor symptoms that frequently emerge before motor onset. These include autonomic dysfunction, cognitive decline, neuropsychiatric disturbances, sleep-related disorders, and sensory deficits. Here, we synthesize current evidence on the anatomical, neurochemical, and network-level mechanisms that drive these symptoms, and we examine how they shape disease progression and clinical heterogeneity. We highlight the limitations of dopamine-centric models and advocate for a framework that treats non-motor symptoms as the disorder’s primary, mechanistically distinct features. We also discuss how emerging technologies—such as multi-omic profiling, artificial intelligence, and network neuroscience—enable earlier identification, stratification of non-motor phenotypes, and the development of precision-based therapeutic strategies. Recognizing non-motor symptoms as central to Parkinson’s disease redefines how the disorder should be diagnosed, studied, and treated.

## 1. Introduction

Parkinson’s disease (PD) has long been defined by its cardinal motor features—tremor, rigidity, and bradykinesia. However, a growing body of evidence shows that non-motor symptoms often precede motor onset by years and contribute more significantly to long-term disability and reduced quality of life. These symptoms—including sensory alterations, autonomic dysfunction, sleep-related disorders, neuropsychiatric features, and cognitive decline—are not ancillary, but integral to PD pathophysiology. Their emergence correlates with dysfunction across multiple neurotransmitter systems, including serotonergic, noradrenergic, and cholinergic networks, extending the scope of PD beyond dopamine depletion in the basal ganglia [1,2,3,4,5,6].

Neuropathological studies have traced the origins of PD-related neurodegeneration to extranigral regions. Lewy bodies, first described in 1912, and later models by Braak and colleagues positioned α-synuclein pathology in the olfactory bulb, the dorsal motor nucleus of the vagus, and autonomic ganglia as early events in disease progression [2,3,4,5,6,7,8]. These observations help explain why hyposmia, REM sleep behavior disorder, and autonomic failure often appear long before motor symptoms. However, not all patients follow Braak’s proposed caudorostral staging. Moreover, α-synuclein burden does not always correlate with neurodegeneration or clinical severity, suggesting that other mechanisms—such as mitochondrial dysfunction, neuroinflammation, and gut–brain axis disruption—also drive disease progression [9,10].

**Figure 1:**
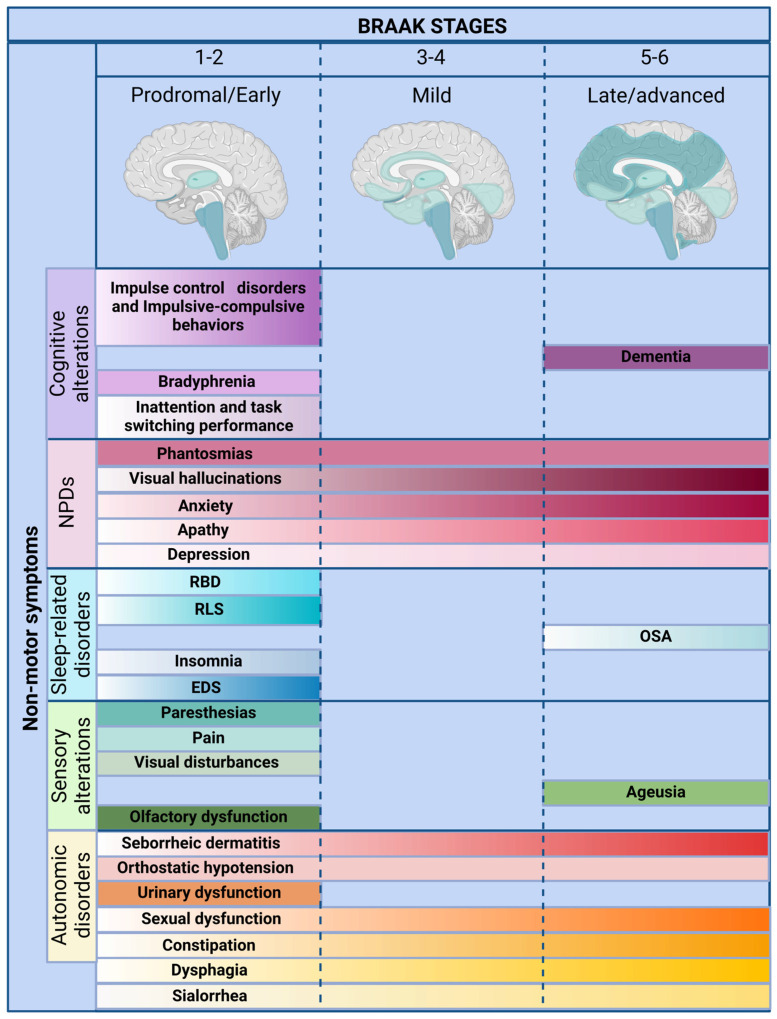
Temporal emergence of non-motor symptoms in Parkinson’s disease relative to Braak staging. The evolving trajectory of non-motor symptoms across Braak stages 1 through 6, aligned with the ascending neuroanatomical propagation of α-synuclein pathology. Symptoms are grouped by domain—autonomic, sensory, sleep-related, neuropsychiatric, and cognitive—and plotted according to their earliest reported onset in prodromal, early, and advanced disease stages. A color gradient represents the intensification of each symptom over time. Early autonomic signs (e.g., constipation, orthostatic hypotension) and REM sleep behavior disorder typically precede motor onset, highlighting their diagnostic and predictive relevance. Mid-stage features such as apathy and visual hallucinations reflect advancing pathology, while cognitive decline and dementia dominate in later stages. This temporal map underscores the value of early symptom recognition for diagnosis, prognosis, and disease stratification. Abbreviations: EDS, excessive daytime sleepiness; NPDs, neuropsychiatric disorders; OSA, obstructive sleep apnea; RBD, REM sleep behavior disorder; RLS, restless leg syndrome. Figure created with BioRender.com, accessed on 5 February 2025. A color gradient represents the intensification of each symptom over time.

**Figure 2:**
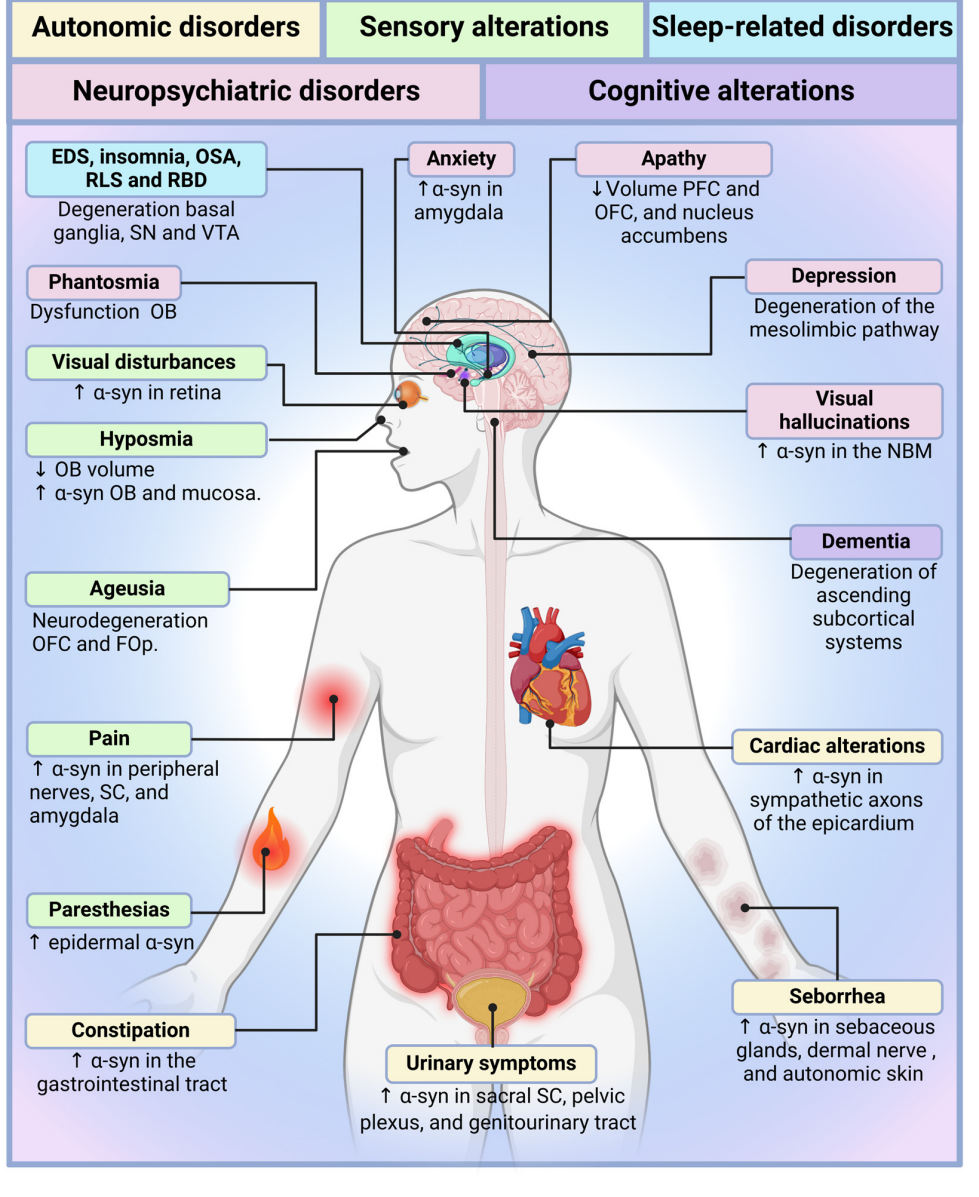
Neuroanatomical substrates and mechanistic underpinnings of non-motor symptoms in Parkinson’s disease. This illustration maps the brain and peripheral regions implicated in non-motor manifestations of Parkinson’s disease, integrating structural and neurochemical changes with clinical symptom domains. α-Synuclein accumulation and neuronal loss in brain stem nuclei (e.g., dorsal motor nucleus of the vagus, locus coeruleus), limbic areas (e.g., amygdala, orbitofrontal cortex), and cholinergic centers (e.g., nucleus basalis of Meynert) disrupt circuits involved in autonomic regulation, emotional processing, arousal, memory, and sleep–wake transitions. Degeneration in the hypothalamic and prefrontal regions contributes to apathy, anxiety, and thermoregulatory failure, while dysfunction in the olfactory bulb, spinal cord, and peripheral autonomic ganglia gives rise to sensory and visceral symptoms. The convergence of central and peripheral pathology reinforces the need to reconceptualize Parkinson’s disease as a network-level, multisystem disorder rather than a focal basal ganglia syndrome. Abbreviations: ↑ = Increased; ↓ = Decreased; α-syn, α-synuclein; EDS, excessive daytime sleepiness; FOp, frontal operculum; NBM, nucleus basalis of Meynert; OB, olfactory bulb; OFC, orbitofrontal cortex; OSA, obstructive sleep apnea; PFC, prefrontal cortex; RBD, REM sleep behavior disorder; RLS, restless leg syndrome; SC, spinal cord; SN, substantia nigra; VTA, ventral tegmental area. Figure created with BioRender.com, accessed on 5 February 2025.

Recognition of the primacy of non-motor symptoms in PD has prompted a reexamination of traditional clinical frameworks. While previous reviews have examined specific symptom domains, few have offered an integrated synthesis grounded in anatomical, neurochemical, and therapeutic perspectives (Figure 1 and Figure 2). This review highlights converging mechanisms across symptom clusters, examines their temporal and pathological trajectories, and assesses how they impact diagnosis and treatment.

We target a broad audience, including medical students, non-specialist physicians, and neuroscientists. We also discuss how emerging technologies—including multi-omic profiling, artificial intelligence, and network-based approaches—may refine patient stratification and therapeutic design. Reframing non-motor symptoms as core disease features, we call for a paradigm shift in PD research and clinical care, emphasizing the need for holistic, multidimensional models.

## 2. Autonomic Dysfunction in Parkinson’s Disease

Autonomic dysfunction is a core and early component of PD, affecting over 90% of patients across multiple physiological systems (see Table 1) [11]. These disturbances frequently precede motor onset stemming from widespread neurodegeneration extending beyond the basal ganglia. α-Synuclein aggregates accumulate in autonomic ganglia and peripheral nerves, disrupting sympathetic and parasympathetic regulation and impairing cardiovascular control, gastrointestinal motility, urinary function, and thermoregulation [12]. Despite its high prevalence and clinical impact, autonomic dysfunction remains underrecognized and poorly managed, often identified only when symptoms become disabling.

Cardiovascular manifestations, including orthostatic hypotension and arrhythmias, elevate fall and cardiovascular risk [13,14]. Gastrointestinal symptoms such as constipation, sialorrhea, and dysphagia frequently appear in the prodromal phase and reflect neurodegeneration in the enteric and parasympathetic systems [13,14]. Urinary dysfunction, anhidrosis, hyperhidrosis, and seborrhea further illustrate the diffuse nature of PD-related autonomic failure. Cutaneous and thermoregulatory disturbances, though commonly dismissed, may offer clinically useful early markers. Collectively, these features highlight the need for early detection and mechanistically informed interventions (Figure 1 and Figure 2) [15].

### 2.1. Sympathetic Autonomic Nervous System

Sympathetic dysfunction in PD typically emerges before motor onset and manifests clinically as orthostatic hypotension, impaired baroreflex sensitivity, and autonomic cardiovascular instability [16]. These features arise from combined central and peripheral neurodegeneration, including loss of preganglionic neurons and postganglionic sympathetic fibers [13]. α-Synuclein pathology in sympathetic ganglia, heart, adrenal tissue, and skin confirms that PD extends far beyond the central nervous system [17,18].

Autonomic structures derived from the neural crest—including the adrenal medulla, sympathetic chain, and spinal ganglia—show marked α-synuclein accumulation, reflecting the multisystem nature of PD [19]. Norepinephrine depletion, especially in cardiac sympathetic fibers, impairs vasoconstriction and contributes to orthostatic hypotension [20]. Pathology studies reveal the selective vulnerability of preganglionic neurons in the thoracic spinal cord and α-synuclein deposition in the celiac ganglion, which controls postganglionic output to visceral organs [10].

Although these mechanisms are increasingly well characterized, therapeutic strategies remain reactive and symptomatic. Clinicians rely on vasopressors and volume-expanding agents to control hypotension, but no disease-modifying approaches have targeted sympathetic neurodegeneration directly. Biomarkers of sympathetic failure, including MIBG cardiac imaging and skin α-synuclein detection, hold diagnostic promise, yet remain underutilized. Despite compelling evidence, sympathetic dysfunction remains peripheral to most PD frameworks, limiting progress in basic and translational domains.

Integrating assessments of autonomic dysfunction—such as 24 h ambulatory blood pressure monitoring, skin sympathetic response testing, and the SCOPA-AUT questionnaire—into the diagnostic and therapeutic frameworks of PD may be helpful for early detection and comprehensive management of non-motor symptoms [12,21,22,23]. These tools facilitate the identification of autonomic impairments that may precede motor symptoms, thereby enabling timely interventions that can improve patient outcomes.

### 2.2. Parasympathetic Autonomic Nervous System

Parasympathetic dysfunction contributes prominently to early gastrointestinal, urological, and cardiovascular symptoms in PD. Delayed gastric emptying, constipation, urinary retention, and chronotropic incompetence are among the earliest indicators of autonomic failure and often precede motor symptoms by several years [24].

The dorsal motor nucleus of the vagus (DMV)—a primary parasympathetic center—is among the first sites to exhibit α-synuclein pathology [9]. Its degeneration disrupts efferent parasympathetic output to the heart, gastrointestinal tract, and bladder, leading to multisystem dysfunction. DMV involvement supports the Braak staging model of caudorostral spread, though its diagnostic and therapeutic value remains underexplored.

Gastrointestinal symptoms arise from α-synuclein pathology in the DMV and enteric nervous system, reinforcing the gut–brain axis hypothesis. Recent studies implicate gut dysbiosis and increased intestinal permeability as potential accelerants of α-synuclein propagation and systemic inflammation. Cardiovascular parasympathetic failure impairs vagal modulation of heart rate and may contribute to arrhythmogenesis and sudden cardiac events [25].

Despite the breadth of symptoms, management remains limited to cholinergic or serotonergic agents and supportive therapies. Dysautonomia receives little attention in clinical trials, and parasympathetic pathology remains poorly integrated into PD treatment algorithms. A shift toward mechanistic interventions and biomarker-guided approaches is essential to move beyond symptom suppression toward precision therapeutics [26].

### 2.3. Sialorrhea and Dysphagia

Sialorrhea and dysphagia are among the most disabling non-motor symptoms of PD, significantly affecting quality of life and increasing morbidity (see Table 1). Sialorrhea affects up to 84% of patients and reflects impaired oral-phase swallowing rather than hypersalivation [27,28,29,30]. Orofacial bradykinesia, α-synuclein accumulation in brain stem nuclei, and cholinergic deficits contribute to reduced clearance of saliva. Dysphagia, affecting up to 87% of patients, progresses alongside motor symptoms and increases the risk of aspiration pneumonia—a leading cause of death in PD [31,32].

Neurodegeneration in the DMV and nucleus ambiguus impairs reflexive and voluntary swallowing [33,34,35,36]. Dopaminergic and cholinergic dysfunction further disrupts motor coordination during the oropharyngeal and esophageal phases. Although dopaminergic therapy may offer partial benefit, it often exacerbates incoordination. Botulinum toxin injections and anticholinergics can relieve sialorrhea, but risk worsening dysphagia. Swallowing rehabilitation, compensatory strategies, and dietary modifications remain central to care, yet long-term efficacy is limited. Multidisciplinary interventions involving speech therapists, neurologists, and nutritionists are essential.

Despite their impact, research into sialorrhea and dysphagia remains limited. These symptoms are not routinely assessed in clinical practice or trials. Targeting early brain stem involvement may offer opportunities for neuroprotective intervention, but such approaches remain unexplored. A more proactive diagnostic framework is required to prevent hospitalizations and complications.

### 2.4. Constipation

Constipation is one of the earliest and most common non-motor symptoms of PD, affecting up to 63% of patients and often emerging decades before motor signs (see Table 1) [37,38]. Early α-synuclein deposition in the enteric nervous system, DMV, and sacral parasympathetic nuclei underpins this symptom’s prominence in prodromal PD [39].

Disruption of cholinergic and serotonergic regulation leads to slowed colonic transit, incomplete evacuation, and dyssynergic defecation [40]. Altered gut microbiota and increased intestinal permeability contribute to systemic inflammation and may promote α-synuclein propagation via the gut–brain axis [41,42]. Small intestinal bacterial overgrowth (SIBO) and gut dysbiosis exacerbate gastrointestinal symptoms and may represent modifiable contributors to disease progression.

Despite its clinical relevance, constipation remains poorly managed. Most treatments—including laxatives, stool softeners, and fiber supplements—target symptoms rather than underlying neuropathology. Prokinetic agents show limited efficacy. A multidisciplinary approach incorporating neurologists and gastroenterologists is essential for optimal care. Constipation should be recognized not only as a quality-of-life issue but also as a potential driver of systemic neurodegeneration.

### 2.5. Neurogenic Sexual Dysfunction

Sexual dysfunction affects approximately 68% of men and 53% of women with PD [43]. Loss of libido, erectile dysfunction in men, and vaginal dryness in women are common manifestations. Neurodegeneration in the hypothalamus, amygdala, and spinal autonomic circuits contributes to disrupted sexual arousal, motivation, and performance (see Table 1) [44,45,46,47].

The mesolimbic dopaminergic pathway and hypothalamic structures, including the medial preoptic area and paraventricular nucleus, undergo early dysfunction in PD. Dopaminergic medications may paradoxically worsen symptoms by inducing impulse control disorders such as hypersexuality [46,48]. Deep brain stimulation of basal ganglia structures can alter sexual behavior, underscoring their regulatory role [49].

Despite the clear neurobiological basis, sexual dysfunction is underdiagnosed due to stigma and limited clinician inquiry. Treatment remains symptomatic, using PDE-5 inhibitors or hormone replacement, with limited mechanistic targeting. A multidisciplinary framework integrating neurology, psychiatry, and sexual health is critical for addressing this overlooked domain.

### 2.6. Urinary Symptoms

Urinary dysfunction is a pervasive, yet often overlooked non-motor symptom of PD, affecting approximately 61% of patients and profoundly impacting quality of life [50,51]. In more severe cases, lower urinary tract symptoms manifest as urgency, frequency, nocturia, incontinence, and urinary retention (see Table 1). These disturbances frequently appear in the premotor phase, reinforcing that PD is a multisystem disorder extending beyond the basal ganglia long before motor symptoms emerge [52]. As the disease progresses, urinary dysfunction correlates with increased motor disability and more extensive dopaminergic denervation, yet the underlying mechanisms remain only partially understood.

The pathophysiology of urinary symptoms in PD involves complex interactions between the central and peripheral nervous systems. The basal ganglia, through its dopaminergic D1-GABAergic direct pathway, plays a crucial role in suppressing involuntary bladder contractions, ensuring appropriate timing of voiding. Dysfunction in this circuit leads to detrusor overactivity, one of the most common urinary disturbances in PD, resulting in urgency and incontinence [52]. However, beyond dopaminergic deficits, neurodegeneration extends to brain regions directly involved in bladder control. The pontine micturition center, which coordinates voluntary voiding, exhibits α-synuclein pathology, as do preganglionic and postganglionic sympathetic neurons, sacral parasympathetic nuclei, and the pelvic plexus [51]. This widespread neurodegeneration disrupts the delicate balance between excitatory and inhibitory control of the bladder, leading to a spectrum of voiding dysfunctions that fluctuate with disease severity.

The presence of α-synuclein aggregates in the genitourinary tract further supports that PD involves peripheral autonomic structures not confined to the central nervous system [53,54]. These pathological changes suggest that bladder dysfunction is not simply a downstream consequence of motor impairment, but a fundamental feature of PD progression. Despite this, urinary symptoms remain underdiagnosed and undertreated, often attributed to ageing rather than being recognized as a direct consequence of neurodegeneration.

Current treatment strategies for urinary dysfunction in PD remain primarily symptomatic [55]. Anticholinergic agents and β3 adrenergic receptor agonists provide some relief by reducing detrusor overactivity, yet cognitive side effects and inconsistent efficacy often limit their effectiveness. Dopaminergic therapies, which are the mainstay of PD treatment, offer little to no improvement in urinary symptoms, highlighting the need for alternative therapeutic approaches that target non-dopaminergic pathways [55]. Non-pharmacological interventions, including pelvic floor therapy and behavioral modifications, are frequently recommended, yet their long-term impact on disease progression remains uncertain [56].

### 2.7. Cardiac Alterations

Autonomic cardiac dysfunction is a common but often underrecognized feature of PD, emerging early in the disease course and progressively worsening as neurodegeneration advances [57]. The imbalance between increased sympathetic activity and decreased parasympathetic regulation leads to significant cardiovascular abnormalities, including heart rate variability, arrhythmias, prolonged P–R and Q–T intervals, and in rare cases cardiomyopathy [58,59]. These alterations not only contribute to increased morbidity but also elevate the risk of sudden cardiac events, underscoring the systemic nature of PD.

The pathophysiology of cardiac dysfunction in PD is driven by widespread autonomic degeneration, characterized by selective loss of sympathetic nerve terminals. Immunohistochemical analyses for tyrosine hydroxylase (TH), a key enzyme in catecholamine synthesis, confirm marked sympathetic denervation of the heart. This loss has been further validated by imaging studies using (123I)-metaiodobenzylguanidine (123I-MIBG) and (18F)-fluorodopa, which demonstrate reduced cardiac norepinephrine uptake, indicative of sympathetic failure [60]. Post-mortem studies reveal α-synuclein aggregates within the epicardial sympathetic axon terminals, with axonal degeneration progressing in a distal-to-proximal manner—first affecting the heart before extending to prevertebral and paravertebral sympathetic ganglia [61].

This pattern of degeneration suggests that cardiac autonomic dysfunction in PD is not merely a secondary consequence of motor impairment or medication side effects, but rather a primary feature of disease pathology [26]. The involvement of the central and peripheral autonomic nervous systems reinforces that PD extends far beyond the dopaminergic system, disrupting regulatory networks that control cardiovascular homeostasis. Despite this, cardiac dysfunction remains underdiagnosed in routine clinical assessments, largely due to the overshadowing focus on motor symptoms.

Despite robust pathology, cardiac dysfunction remains underdiagnosed. Treatment is symptomatic, using beta-blockers or volume expanders [62,63]. No therapies target the underlying autonomic degeneration. Greater integration of cardiovascular assessments into PD care is needed.

### 2.8. Orthostatic Hypotension

Orthostatic hypotension (OH) is a debilitating and often underdiagnosed cardiovascular manifestation of PD, affecting approximately 30% of patients. Defined by a drop of more than 20 mmHg in systolic blood pressure or 10 mmHg in diastolic pressure within three minutes of standing, OH significantly impairs daily function, increasing the risk of dizziness, fatigue, syncope, falls, and injury [64,65]. Unlike primary hypotensive disorders, neurogenic OH in PD arises from a fundamental breakdown in autonomic regulation, reflecting widespread sympathetic denervation and baroreflex failure [66].

The underlying pathophysiology of OH in PD stems from impaired norepinephrine release, leading to deficient vasoconstriction and a subsequent failure to maintain blood pressure upon standing [65,67]. Studies indicate this dysfunction is primarily driven by sympathetic neural degeneration rather than baroreceptor failure, distinguishing Parkinson’s-related OH from other forms of dysautonomia [68,69]. The depletion of norepinephrine at both central and peripheral levels compromises vascular tone, exacerbating cerebral autoregulation deficits that further contribute to orthostatic intolerance. The insular cortex, a critical autonomic control region, also shows significant Lewy body accumulation in PD, correlating with the severity of OH and the overall progression of autonomic dysfunction [66].

Twenty-four-hour ambulatory blood pressure monitoring may be a valuable tool for detecting cardiovascular autonomic dysfunction, including abnormal circadian blood pressure patterns such as reverse dipping, which are associated with increased risk of adverse clinical events [21,70].

Despite its high prevalence and profound impact on patient well-being, OH remains inadequately managed in PD [71]. Symptomatic treatments include fludrocortisone, midodrine, droxidopa, and lifestyle modifications [72]. However, these interventions do not halt neurodegeneration. Targeted therapies remain lacking, underscoring the need for autonomic biomarkers in early disease.

### 2.9. Seborrhea and Seborrheic Dermatitis

Seborrhea and seborrheic dermatitis are common non-motor manifestations in PD, affecting between 52% and 59% of patients and frequently appearing in early disease stages [73]. Characterized by erythema, greasy scaling, and increased sebum production, these conditions reflect underlying autonomic dysregulation rather than primary dermatologic disease. Sebaceous gland activity is modulated by dopaminergic signaling, and degeneration of basal ganglia circuits disrupts this regulatory control [74].

Malassezia yeast colonization contributes to inflammation and barrier disruption; however, excessive sebum production and altered skin microbiota are likely consequences of neurodegenerative processes [75,76]. Histopathological studies reveal α-synuclein aggregates in dermal autonomic fibers and sebaceous glands, suggesting that cutaneous involvement is part of systemic PD pathology [76]. These changes offer a potential avenue for biomarker development. Indeed, skin biopsies detecting phosphorylated α-synuclein in peripheral nerves are under investigation as early diagnostic tools.

Despite their high prevalence, seborrheic manifestations are rarely integrated into PD assessment [74,77]. Treatment typically involves antifungals and corticosteroids, offering temporary symptom control without addressing the neurobiological substrate. Collaborative care between neurologists and dermatologists is needed to improve diagnosis and uncover therapeutic opportunities.

### 2.10. Anhidrosis/Hyperhidrosis

Sweating disturbances are frequently reported, but underexamined in PD. Hyperhidrosis (excessive sweating) typically affects the upper body, while anhidrosis (reduced sweating) is more common in the extremities, resulting in an asymmetric thermoregulatory profile [78,79]. These disturbances occur independently of motor symptom severity and reflect complex autonomic dysfunction.

Central mechanisms, including hypothalamic dysregulation and α2 adrenergic receptor dysfunction, contribute to abnormal sympathetic output. Peripherally, progressive pre- and postganglionic sympathetic innervation loss disrupts sudomotor function [80,81]. The resulting thermoregulatory imbalance exacerbates orthostatic symptoms, fatigue, and discomfort, impairing daily activities and sleep quality.

Treatment remains empirical. Anticholinergic agents and botulinum toxin injections can reduce hyperhidrosis, but risk side effects [82,83,84]. Anhidrosis lacks effective therapy. No interventions target the underlying autonomic neurodegeneration. These symptoms may serve as early markers of systemic dysfunction and merit further investigation.

### 2.11. Thermoregulatory Alterations

Thermoregulatory dysfunction is a clinically significant, yet underrecognized non-motor feature of PD. Symptoms include cold intolerance, paradoxical sweating, and episodes of hypothermia, which can occur even in normothermic environments [85,86]. Hypothermia (<35 °C) poses serious systemic risks, including arrhythmias and altered mental status.

The hypothalamus orchestrates thermoregulatory control through modulation of vasomotor tone, sweat gland activity, and metabolic rate. PD-related degeneration disrupts these mechanisms, impairing heat dissipation and conservation [85]. Sympathetic dysfunction reduces vasoconstrictive response and heat generation, while parasympathetic hyperactivity impairs recovery from cold exposure [80,87].

Sympathetic neurograms and heart rate variability studies confirm diminished autonomic responsiveness in PD. These impairments correlate with disease duration and severity, suggesting progressive autonomic decline. Despite the clinical relevance, no targeted therapies have addressed thermoregulatory dysfunction. Management focuses on behavioral adaptations and environmental controls.

Recognition of thermoregulatory abnormalities as intrinsic to PD pathology—not incidental complications—is essential for improving patient safety and quality of life. Greater mechanistic understanding and biomarker integration are needed to inform preventive and therapeutic strategies.

## 3. Sensory Alterations in Parkinson’s Disease

Sensory dysfunction is an increasingly recognized, but still underappreciated aspect of PD. These alterations—including hyposmia, ageusia, visual impairment, pain, and paresthesias—often precede motor onset and affect up to 90% of patients [88,89]. Their high prevalence and early appearance suggest a central role in PD pathophysiology (see Table 2). Despite this, most are underdiagnosed and undertreated, reflecting the persistent dominance of a motor-centric clinical model. Here, we examine the anatomical, molecular, and clinical underpinnings of PD-related sensory dysfunction, emphasizing their diagnostic, prognostic, and therapeutic relevance (Figure 1 and Figure 2).

### 3.1. Hyposmia

Olfactory dysfunction is one of the most well-established non-motor symptoms of PD, first recognized over three decades ago as a frequent yet overlooked aspect of the disease [90,91,92]. Hyposmia, characterized by a diminished sense of smell, affects between 75% and 90% of patients and often precedes the onset of motor symptoms by several years. This strong temporal association makes olfactory impairment one of the most reliable early biomarkers of PD, with individuals experiencing hyposmia exhibiting a 3.84-fold increased risk of developing the disorder [93,94,95,96,97].

Hyposmia in PD is not merely an isolated sensory impairment, but rather a reflection of widespread neurodegeneration. The severity of olfactory dysfunction correlates with cognitive decline, depression, anxiety, and REM sleep behavior disorder, suggesting a shared pathophysiological mechanism underlying these non-motor symptoms [98,99,100,101]. The neuropathological hallmark of PD—Lewy pathology—originates in the olfactory bulb and spreads along neural pathways, leading to neuronal loss and reduced olfactory bulb volume. This process extends to limbic structures, including the amygdala and hippocampus, further implicating olfactory dysfunction as a harbinger of broader neurodegeneration [9,102,103].

Identifying α-synuclein aggregates in olfactory mucosal receptor neurons has raised the prospect of using nasal swabs as a potential diagnostic tool for PD (see Table 2). Although preliminary studies support the feasibility of this approach, additional research is needed to refine its sensitivity and specificity before it can be implemented in routine clinical practice [104,105]. Beyond its association with Lewy pathology, olfactory dysfunction in PD is also linked to disruptions in dopaminergic transmission. The nigrostriatal dopaminergic system, known for its role in motor function, also modulates sensory processing, including olfaction. Dopaminergic denervation correlates with the severity of hyposmia, with lower dopamine transporter activity in striatal regions corresponding to reduced olfactory performance [98,106]. Given that up to 80% of dopaminergic neurons are lost before the appearance of motor symptoms, olfactory dysfunction may provide a crucial window for early intervention [107].

Despite its predictive value, hyposmia lacks specificity and is rarely assessed in routine practice [108]. Standardized olfactory testing could enhance early diagnosis, but implementation remains limited. No established therapies exist to reverse olfactory decline. The absence of targeted interventions reflects a broader neglect of sensory symptoms in PD.

### 3.2. Ageusia

Ageusia, the loss of taste perception, is a frequently overlooked sensory deficit in PD, often dismissed as a secondary effect of olfactory dysfunction rather than a distinct neurological impairment (see Table 2). However, evidence suggests that ageusia results from independent neuroanatomical and molecular abnormalities, making it an essential but underrecognized component of the non-motor symptom spectrum. Prevalence estimates vary widely, ranging from 9% to 54%, depending on study methodology and disease stage [109,110,111,112]. Given its significant impact on appetite, nutrition, and overall quality of life, the failure to adequately assess and manage ageusia in PD reflects a broader neglect of sensory dysfunction in clinical practice.

Lewy pathology affects gustatory processing areas, including the orbitofrontal cortex, insula, and sensory integration hubs [109,113]. Cholinergic degeneration disrupts acetylcholine-dependent pathways, impairing taste perception and sensory integration [114,115]. Cognitive decline exacerbates these deficits by altering network connectivity across taste-related circuits.

At the molecular level, downregulation of taste receptor genes has been observed in PD models, suggesting that ageusia arises from combined neurodegenerative and genetic mechanisms [116]. Despite these findings, no diagnostic protocols or targeted treatments exist for ageusia in PD. Clinical oversight of this symptom reflects an outdated framework that minimizes sensory dysfunction.

### 3.3. Visual Disturbances

Visual impairment affects over 90% of patients with PD and ranges from reduced contrast sensitivity to hallucinations [117,118,119]. These deficits impact mobility, safety, and cognitive performance. Despite their prevalence, visual symptoms are rarely prioritized in PD care (see Table 2).

Visual dysfunction in PD arises from both cortical and retinal degeneration. Lewy pathology affects visual cortices and correlates with visuospatial and perceptual deficits [119,120]. Retinal dopaminergic amacrine cells degenerate early, impairing contrast sensitivity and color discrimination [121,122,123,124].

Retinal imaging via optical coherence tomography (OCT) reveals thinning of the retinal nerve fiber layer and microvascular alterations, correlating with disease severity [125,126,127]. These biomarkers may enable earlier diagnosis and disease monitoring, but clinical integration remains limited.

Therapeutic options are underdeveloped [128]. Dopaminergic therapy yields inconsistent benefits. Visual aids and rehabilitation strategies are underused. Diagnostic opportunities and symptom management will continue to lag without integrating visual assessment into PD protocols.

### 3.4. Pain

Pain is a highly prevalent, yet frequently underestimated non-motor symptom of PD, affecting between 20% and 98% of patients depending on study methodology and diagnostic criteria [129,130,131]. For many, pain is not merely a secondary complication, but a defining feature of the disease, often preceding the onset of motor symptoms by several years (see Table 2). The nature of pain in PD is heterogeneous, encompassing musculoskeletal pain linked to rigidity and postural abnormalities, dystonic pain arising from involuntary muscle contractions, and neuropathic or radicular pain indicative of peripheral nerve involvement [4,129]. Despite its impact on daily life, pain remains underdiagnosed mainly and poorly managed, highlighting the need for a more integrated approach to its assessment and treatment.

The pathophysiology of pain in PD is multifaceted, involving both central and peripheral mechanisms. Lewy pathology extends beyond the basal ganglia to regions directly involved in pain processing, including the spinal cord, amygdala, periaqueductal gray, and nucleus accumbens. Neurodegeneration in these areas alters nociceptive modulation, increasing pain sensitivity and disrupting the endogenous analgesic system [132]. Additionally, PD affects non-dopaminergic structures such as the subthalamic nucleus, anterior cingulate cortex, and insular cortex—regions implicated in pain perception and emotional processing—further amplifying the subjective experience of pain [133,134,135].

Dysregulation of pain-modulating neurotransmitters is another key contributor to heightened pain sensitivity in PD. Beyond dopamine, disruptions in norepinephrine and serotonin transmission impair descending pain inhibition, intensifying nociceptive signaling and altering the brain’s ability to regulate pain perception [135,136]. Furthermore, neuroinflammation is critical, as elevated levels of proinflammatory cytokines contribute to peripheral and central sensitization, exacerbating pain symptoms [137,138]. These overlapping mechanisms suggest that pain in PD is not merely a consequence of motor dysfunction, but a fundamental component of disease pathology, warranting targeted therapeutic strategies.

Despite its high prevalence, no definitive biomarkers have been identified to correlate pain severity with specific neuropathological changes in PD [139]. While some patients experience pain long before motor symptoms emerge, the mechanisms underlying this early manifestation remain poorly understood. Identifying reliable biomarkers and delineating the neurochemical pathways involved in Parkinson’s-related pain is critical for developing precision-targeted therapies.

Current treatment approaches remain largely symptomatic, relying on pharmacological and non-pharmacological interventions. However, conventional dopaminergic therapy provides inconsistent relief, underscoring the need for alternative strategies that address the non-dopaminergic contributions to pain processing. Neuromodulators and physical therapies offer partial relief, but lack disease-modifying potential. Recognizing pain as a primary neurodegenerative symptom is critical for targeted research and comprehensive care.

### 3.5. Paresthesias

Paresthesias—tingling, numbness, or burning sensations—affect approximately 40% of PD patients [140]. These symptoms are frequently misattributed to aging or comorbid neuropathy. However, α-synuclein pathology in cutaneous and peripheral nerves suggests a direct neurodegenerative basis (see Table 2) [141,142,143,144].

Small-fiber neuropathy, Schwann cell dysfunction, and autonomic denervation contribute to altered somatosensory signaling [145,146,147,148]. Loss of epidermal nerve fibers correlates with disease severity. Autonomic impairment alters pain thresholds and temperature perception, compounding paresthetic symptoms.

Despite their impact, paresthesias remain underdiagnosed. No standard diagnostic tools or therapeutic guidelines exist. Current treatment is symptomatic and often inadequate. Recognition of paresthesias as a core feature of PD could stimulate mechanistic research and improve patient outcomes.

## 4. Sleep-Related Disorders in Parkinson’s Disease

Sleep disturbances are among the most prevalent and disabling non-motor symptoms of PD, affecting between 60% and 98% of patients [149]. These disorders impair cognitive function, exacerbate mood instability, and worsen motor symptoms, creating a feedback loop that accelerates neurodegeneration. Despite their frequency, sleep-related symptoms remain underdiagnosed and undertreated, frequently overshadowed by the emphasis on motor impairment in clinical assessments (see Table 3).

The spectrum of PD-related sleep disturbances is broad, encompassing insomnia, excessive daytime sleepiness, REM sleep behavior disorder (RBD), restless leg syndrome (RLS), obstructive sleep apnea (OSA), and nocturia. Many of these manifestations emerge during the prodromal phase, well before motor symptoms, positioning them as early biomarkers of neurodegeneration [149]. Their diagnostic value and impact on disease trajectory make sleep dysfunction an essential, yet overlooked component of PD pathophysiology (Figure 1 and Figure 2).

### 4.1. REM Sleep Behavior Disorder

RBD is characterized by the loss of muscle atonia during REM sleep, leading to dream enactment behaviors that may result in self-injury or harm to bed partners. It is one of the most specific prodromal markers of synucleinopathies. Longitudinal studies show that 80%–90% of patients with idiopathic RBD develop PD, dementia with Lewy bodies, or multiple system atrophy within 10–15 years [149].

The pathophysiology of RBD implicates early degeneration in the pontine tegmentum, including the sublaterodorsal nucleus, and magnocellular reticular formation. These brain stem circuits regulate REM atonia, and their dysfunction precedes motor decline. RBD also correlates with more rapid cognitive deterioration, hallucinations, and autonomic instability, supporting its role as a marker of a more malignant PD phenotype.

Despite its predictive value, RBD remains underdiagnosed. Polysomnography is required for definitive diagnosis, yet few centers implement routine screening. Clonazepam and melatonin offer partial symptomatic relief, but no disease-modifying interventions exist. Further research into RBD-associated neurodegeneration could improve early diagnosis and therapeutic targeting.

### 4.2. Insomnia and Sleep Fragmentation

Insomnia affects up to 80% of individuals with PD and contributes significantly to cognitive decline, fatigue, and emotional dysregulation [149,150,151]. Sleep-onset and maintenance difficulties arise from nocturnal motor symptoms, including rigidity, tremor, and dystonia, but also reflect intrinsic disruption of sleep-regulating circuits.

Neurodegeneration in serotonergic and noradrenergic brain stem nuclei impairs sleep–wake stability, while degeneration of the ventrolateral preoptic nucleus disrupts sleep-promoting mechanisms. Fragmented sleep architecture and reduced REM sleep duration are common, often compounded by anxiety and depression.

Despite its burden, insomnia in PD is often addressed using sedative-hypnotics with limited benefit and potential adverse effects. Cognitive behavioral therapy for insomnia (CBT-I) and light therapy have demonstrated efficacy, but remain underused. Recognition of insomnia as a core manifestation of PD is essential for advancing treatment beyond symptom suppression.

### 4.3. Excessive Daytime Sleepiness

Excessive daytime sleepiness (EDS) occurs in up to 76% of PD patients and worsens with disease progression and dopaminergic therapy, particularly dopamine agonists [149]. EDS impairs attention, increases accident risk, and contributes to social withdrawal.

The etiology of EDS is multifactorial. Neurodegeneration in the locus coeruleus, tuberomammillary nucleus, and orexin-producing neurons in the lateral hypothalamus impairs wakefulness regulation. Medications exacerbate this dysfunction, and nocturnal sleep fragmentation compounds daytime fatigue.

Management of EDS remains limited to reducing sedating medications and promoting sleep hygiene. Modafinil and other wake-promoting agents offer partial benefits, but are inconsistently effective. Greater mechanistic understanding is needed to guide future interventions.

### 4.4. Restless Leg Syndrome and Obstructive Sleep Apnea

RLS is characterized by an urge to move the legs and uncomfortable sensations that worsen at night. It disrupts sleep quality and affects up to 20% of PD patients [149]. The pathogenesis remains unclear, but may involve dopaminergic and iron homeostasis abnormalities in the spinal cord and basal ganglia.

OSA affects approximately 20%–60% of individuals with PD and contributes to fragmented sleep, intermittent hypoxia, and oxidative stress. It has been linked to cognitive decline and may accelerate neurodegeneration. Polysomnographic evaluation and continuous positive airway pressure (CPAP) use remain underutilized.

### 4.5. Mechanisms and Implications for Disease Progression

Sleep-related disorders in PD arise from degeneration of the brain stem, hypothalamic, and cortical structures regulating sleep–wake transitions. Dopaminergic loss in the substantia nigra and ventral tegmental area destabilizes circadian and behavioral arousal. Early α-synuclein pathology in the raphe nuclei and locus coeruleus impairs serotonergic and noradrenergic tone, essential for REM and non-REM stability [9,10,149].

The flip-flop switch model highlights the role of cholinergic–monoaminergic interactions in sleep control, which are dysregulated in PD [149]. Additionally, the glymphatic system—responsible for clearing misfolded proteins during deep sleep—is compromised in PD, potentially promoting α-synuclein accumulation and disease progression [152]. Circadian dysfunction further exacerbates symptoms through impaired melatonin secretion, hypothalamic disruption, and retinal pathway degeneration [149].

### 4.6. Clinical Management and Future Directions

Current treatments for PD-related sleep disturbances are primarily symptomatic. Melatonin, sedatives, and dopaminergic agents offer partial relief, but often introduce new complications. Non-pharmacological approaches, including CBT-I, light therapy, and CPAP for OSA, are evidence-based, yet underutilized.

There is an urgent need to integrate sleep assessment into routine PD care. Personalized treatment strategies based on sleep phenotyping and early biomarker screening (e.g., RBD) could facilitate timely interventions and improve long-term outcomes. Targeting sleep dysfunction as a modifiable contributor to neurodegeneration could shift therapeutic goals from symptom management to disease modification.

Sleep disturbances are not merely downstream effects of PD, but integral components of its pathology. Addressing them requires a paradigm shift in research, clinical assessment, and care delivery—placing sleep at the forefront of PD management.

## 5. Neuropsychiatric Manifestations in Parkinson’s Disease

Neuropsychiatric symptoms are increasingly recognized as core components of PD, often emerging in the prodromal phase and exerting a profound influence on patient quality of life and disease progression [153]. Depression, anxiety, apathy, visual hallucinations, and phantosmia disrupt emotional regulation, cognitive stability, and social functioning, yet they remain underdiagnosed and inadequately treated (see Table 4). These symptoms are not reactive responses to chronic illness, but result from neurodegenerative changes in mesolimbic, serotonergic, cholinergic, and cortical circuits [154,155]. Recognizing neuropsychiatric dysfunction as integral to PD pathophysiology is essential for advancing timely diagnosis, improving patient outcomes, and developing mechanism-based interventions (Figure 1 and Figure 2).

### 5.1. Depression

Depression is one of the most common neuropsychiatric symptoms of PD, affecting between 35% and 45% of patients (see Table 4). It may present at any stage of the disease, frequently emerging before the onset of motor dysfunction and persisting throughout disease progression [156]. Unlike primary depressive disorders, Parkinson’s-related depression is often characterized by irritability, anhedonia, cognitive slowing, and profound apathy rather than overt sadness, complicating its recognition and differentiation from other non-motor symptoms. Sleep disturbances, fatigue, and psychomotor retardation further contribute to functional impairment, exacerbating the overall disease burden [156,157].

The underlying pathophysiology of Parkinson’s-related depression extends beyond dopamine depletion, implicating widespread neurodegeneration in key mood-regulating structures. Structural imaging studies reveal significant gray matter atrophy in the bilateral thalami and amygdalae, regions critical for emotional processing, while cortical gyrification deficits in the frontal and parietal lobes correlate with depressive severity [158]. Braak’s staging suggests that early degeneration of the mesolimbic dopaminergic system and noradrenergic neurons in the locus coeruleus and serotonergic neurons in the raphe nucleus underlies mood dysregulation in PD [9,10]. However, serotonin transporter reductions do not consistently correlate with depression severity, raising questions about additional mechanisms contributing to these symptoms [159]. Interestingly, increased SNCA gene expression, which encodes α-synuclein, has been observed in patients with Parkinson’s-related depression, suggesting a molecular link between neurodegeneration and mood disorders [160].

However, traditional antidepressants yield inconsistent results in PD. Dopaminergic agents such as pramipexole show promise, but are limited by behavioral side effects [161]. Despite their efficacy, cognitive behavioral therapy, transcranial stimulation, and structured exercise remain underused. A greater focus on tailored, mechanistically informed treatments is needed.

### 5.2. Apathy

Apathy, a profound lack of motivation that is independent of mood, consciousness, or cognition, is one of the most disabling non-motor symptoms of PD [162]. Affecting between 40% and 52% of patients, apathy often emerges early in the disease course with or without coexisting depression or anxiety, yet remains significantly underrecognized and undertreated [163,164]. It manifests in three domains—behavioral and cognitive inertia, emotional blunting, and diminished social interaction—each contributing to a progressive disengagement from daily activities and exacerbating functional decline (see Table 4). Apathy is diagnosed when these deficits persist for at least four weeks and impair at least two domains, making it a critical, yet frequently overlooked determinant of disease burden [165,166].

The impact of apathy extends far beyond motivation deficits, profoundly influencing cognition, emotional well-being, and survival. Patients with apathy exhibit more significant cognitive impairment, more severe depressive symptoms, and an increased risk of mortality compared to those without these symptoms. While early-onset apathy in PD is often associated with concurrent depression, its later emergence signals a heightened risk of cognitive decline and progression to dementia [167,168]. This evolution from a motivational deficit to a harbinger of cognitive deterioration highlights its potential role as a biomarker of disease progression.

Neuroimaging reveals disrupted connectivity in the mesocorticolimbic network, including the orbitofrontal cortex and nucleus accumbens [169,170,171]. Biomarker studies suggest oxidative stress, iron accumulation, and α-synuclein oligomers may exacerbate motivational dysfunction [172,173].

Standard dopaminergic therapies provide limited benefit, and few studies specifically target apathy [174]. Emerging evidence supports structured behavioral interventions and tailored neurostimulation protocols. Given its predictive value for cognitive decline, apathy warrants early recognition and integrated management.

### 5.3. Anxiety

Anxiety is a frequent and often debilitating non-motor symptom of PD characterized by excessive worry, an exaggerated perception of threat, and diminished coping mechanisms (see Table 4). Affecting approximately 31% of patients, anxiety in PD is not merely a secondary reaction to diagnosis, but an intrinsic feature of the disease, emerging as early as the prodromal phase and persisting throughout disease progression [175]. Unlike primary anxiety disorders, Parkinson’s-related anxiety exhibits a unique profile, with generalized anxiety disorder, social phobia, and panic disorder being the most prevalent manifestations [175]. The presence of anxiety accelerates disease progression, exacerbates motor dysfunction, interferes with treatment response, and increases overall mortality risk [176]. Despite its profound impact on patients’ quality of life, anxiety remains largely underdiagnosed and undertreated, often dismissed as a reactive component of living with a neurodegenerative disorder.

The emergence of anxiety in PD is closely tied to structural and functional alterations in key neural circuits. Neuroimaging studies reveal significant atrophy in the left amygdala, frontocingulate cortex, and parietal regions and heightened connectivity within the fear circuit and salience network [177]. These alterations disrupt emotional regulation and threat perception, likely contributing to the heightened anxiety response observed in PD. Furthermore, α-synuclein pathology extends beyond the dopaminergic system, with accumulating evidence linking α-synuclein deposits in the amygdala to anxiety symptoms. Elevated erythrocytic α-synuclein levels have been detected in Parkinson’s patients with anxiety, reinforcing its potential role in disease pathophysiology [178,179]. Beyond neurodegeneration, oxidative stress and systemic inflammation have also been implicated as key contributors to Parkinson’s-related anxiety, suggesting that peripheral immune dysfunction may play a previously underestimated role in neuropsychiatric symptoms [180].

Despite its impact, anxiety is underdiagnosed [181]. SSRIs and benzodiazepines have inconsistent efficacy and potential side effects. Behavioral therapies, including CBT and mindfulness, offer benefits, but are rarely implemented. Stratified approaches based on imaging and biomarker profiles could enhance diagnostic precision and treatment outcomes.

### 5.4. Visual Hallucinations

Visual hallucinations affect 27%–50% of individuals with PD and often indicate progression to cognitive impairment [182,183,184,185,186,187]. Minor hallucinations may precede structured, distressing perceptual disturbances. Their early emergence correlates with cognitive decline and reduced quality of life (see Table 4).

Cholinergic and GABAergic deficits in the ventral visual stream and visual cortex disrupt sensory gating [188,189]. Structural connectivity abnormalities involving the lateral geniculate nucleus, thalamus, and prefrontal cortex further impair perceptual integration [190,191,192,193].

While dose reduction of dopaminergic agents may reduce hallucinations, it risks worsening motor symptoms [194]. Cholinesterase inhibitors show partial benefit. Future research must prioritize predictive biomarkers and preventive strategies to reduce hallucination-related morbidity.

### 5.5. Phantosmia

Phantosmia, the perception of odors without an external source, is reported in up to 18% of patients with PD and may co-occur with other hallucinations [195,196,197,198]. Unlike hyposmia, phantosmia does not correlate with measured olfactory loss, suggesting a distinct pathophysiological basis (see Table 4).

Disruption of predictive coding networks in the olfactory bulb and anterior olfactory nucleus likely contributes to phantosmia [199]. No direct link to Lewy pathology has been established, complicating diagnostic efforts.

Management remains empirical. Further investigation is required to determine whether phantosmia represents a unique prodromal marker or reflects broader cortical sensory dysfunction. Standardized assessment tools and longitudinal studies are needed.

## 6. Cognitive Dysfunction in Parkinson’s Disease

Cognitive dysfunction is a pervasive non-motor feature of PD, with impairments in executive function, attention, and processing speed frequently emerging early in the disease course emerging early and progressively worsening as the disease advances (see Table 5) [200]. Approximately 27% of untreated patients already exhibit cognitive deficits, and prevalence approaches 93% in advanced stages [161,200]. These deficits reflect progressive dysfunction in frontostriatal and frontoparietal networks, beginning in the caudate nucleus and expanding to involve the mesocorticolimbic and thalamocortical circuits [201,202,203,204,205].

Cognitive symptoms disrupt daily functioning, interfere with motor control, and accelerate dependence and institutionalization [201,202,203,204,205,206,207,208]. Despite their clinical relevance, cognitive changes are often misattributed to aging, delaying diagnosis and intervention. Structured cognitive screening and early stratification of cognitive phenotypes remain underutilized in PD care (Figure 1 and Figure 2).

### 6.1. Inattention and Task-Switching Performance

Attentional deficits affect over 20% of drug-naïve PD patients, often preceding motor symptoms [209,210,211]. These impairments hinder goal-directed behavior, impair task switching, and reduce adaptive flexibility. Dopaminergic depletion in mesocortical and frontoparietal circuits underlies reduced top-down and bottom-up attentional control [212,213,214].

Neurophysiological studies reveal abnormal alpha and gamma-band oscillatory activity and disrupted dorsal attention network connectivity [215,216,217,218,219]. Lower-order attentional processes rely on corticostriatal dopamine, while higher-order attentional shifts depend on noradrenergic and serotonergic transmission, suggesting the need for multimodal therapies [220,221,222].

Treatment remains empirical. While dopamine replacement may partially improve attentional performance, patient variability and potential cognitive side effects limit its utility. Cognitive training and stimulant-based therapies are under investigation, but lack standardization. Accurate differentiation between attentional dysfunction and global cognitive decline is essential for early, targeted intervention.

### 6.2. Bradyphrenia

Bradyphrenia—slowing thought processes and reduced cognitive flexibility—is a hallmark of subcortical cognitive dysfunction in PD [201,223]. It contributes to diminished adaptability and poor decision-making, often without overt memory impairment. White matter changes and disrupted noradrenergic signaling have been implicated in its pathophysiology [201,203].

Cognitive assessments frequently detect slowed reaction times and mental inflexibility in patients without dementia, highlighting the need for sensitive diagnostic tools [224,225,226,227]. Computational modeling suggests that bradyphrenia parallels bradykinesia, with retained memory, but impaired strategy shifting and sensorimotor adaptation [225].

Levodopa shows inconsistent benefits and may worsen cognitive efficiency in some individuals [228]. Non-dopaminergic targets—particularly noradrenergic and cholinergic pathways—warrant further exploration. Improved characterization of bradyphrenia is essential to distinguish it from coexisting depression, apathy, or fatigue.

### 6.3. Dementia

PD dementia (PDD) affects 20%–40% of patients and develops in up to 80% over time [229]. Mild cognitive impairment in early PD is a strong predictor of dementia, especially in patients with older age, akinetic-rigid phenotype, hallucinations, or cardiovascular comorbidities [229,230].

PDD involves progressive dysfunction of cortico–striato–thalamo–cortical loops, manifesting as executive, visuospatial, and attentional impairments, often accompanied by hallucinations, delusions, and apathy [231,232,233]. While α-synuclein pathology predominates, co-pathologies such as tau, amyloid-β, and TDP-43 contribute to cognitive decline and variability in clinical phenotype [234,235].

Additional contributors include synaptic dysfunction, neuroinflammation, and ageing-related lesions [236,237]. Despite these insights, no approved treatments delay MCI conversion to dementia. Cholinesterase inhibitors provide modest symptomatic benefits, but precision-targeted therapies remain elusive. Future strategies must incorporate biomarker-driven phenotyping and predictive modeling to personalize interventions.

### 6.4. Impulse Control Disorders and Impulsive-Compulsive Behaviors

Impulse control disorders (ICDs) affect 6%–45% of PD patients, particularly those treated with dopamine agonists [238,239,240,241]. Early-onset PD, male sex, and premorbid addictive traits increase susceptibility. ICDs encompass behaviors such as pathological gambling, compulsive shopping, hypersexuality, and binge eating, leading to significant social and functional disruption [242,243,244].

The pathophysiology involves mesolimbic dopaminergic dysregulation, with contributions from opioid and serotonergic circuits [245,246]. Functional imaging demonstrates enhanced reward sensitivity and impaired inhibitory control. Dopamine agonists potentiate impulsive decision-making and bias reward-based learning [247,248,249].

Reducing dopamine agonist dosage remains first-line management, but often compromises motor control. Cognitive behavioral therapy and novel approaches such as opioid antagonists and neuromodulation are under investigation. Biomarker-guided risk prediction and individualized treatment models will be essential for balancing motor and behavioral outcomes.

## 7. Emerging Technologies and the Future of Personalized Care

Despite decades of research, PD is still predominantly diagnosed based on motor symptoms that appear late in the disease course—by which time significant and irreversible neurodegeneration has occurred. This clinical inertia persists despite overwhelming evidence that non-motor symptoms, including REM sleep behavior disorder, constipation, hyposmia, depression, and anxiety, can precede motor onset for years or even decades. Nevertheless, these early warning signs remain largely ignored in diagnostic frameworks, risk stratification tools, and therapeutic trials. The continued neglect of these symptoms represents a missed opportunity and a critical failure of the field to evolve beyond outdated staging models and symptomatic care.

Emerging technologies—particularly multi-omic profiling, artificial intelligence (AI), and systems-level brain network modeling—potentially shift this paradigm fundamentally. Rather than treating PD as a single, dopaminergic disorder with a uniform trajectory, these tools support a more accurate characterization of disease heterogeneity, enable the identification of prodromal subtypes, and provide insight into mechanisms underlying non-motor burden.

Multi-omic approaches have begun to reveal molecular fingerprints associated with non-motor-dominant PD phenotypes, yet methodological inconsistency, small cohorts, and the absence of standardized biomarker validation pipelines hinder their translation into clinical practice. Despite these limitations, multi-omics provides a foundation for redefining disease subtypes at the molecular level—something urgently needed if personalized interventions are to be developed [250,251,252,253,254].

AI and machine learning offer unprecedented capacity to integrate large, multidimensional datasets, including longitudinal digital biomarkers from wearable sensors, sleep physiology, neuroimaging, and electronic health records. These algorithms have already successfully detected subtle, preclinical motor and non-motor changes. However, clinical deployment has been slow and regulatory frameworks have yet to catch up. Without rigorous validation and representative datasets, AI risks amplifying bias rather than resolving uncertainty [255,256,257].

Network neuroscience reframes PD as a disorder of dynamic, large-scale brain circuits rather than isolated nigral degeneration. Disrupted connectivity in frontostriatal, limbic, and brain stem networks has been linked to depression, apathy, autonomic failure, and cognitive decline—symptoms that drive disability, but are poorly captured by conventional motor scales. Although still in its infancy, this approach offers a systems-level lens to understand phenotypic variability and treatment response [258,259,260,261,262].

The promise of these technologies lies not in their novelty, but in their capacity to disrupt a model of care that has stagnated. Early identification of PD based on non-motor symptoms is not merely a research priority, but a clinical and ethical imperative. However, the field continues to design trials that exclude early-stage or non-motor-dominant patients, rely on scales developed decades ago, and treat emerging tools as experimental add-ons rather than diagnostic essentials.

To realize the potential of precision neurology, the next phase of PD research must reject uniformity, embrace heterogeneity, and prioritize the full spectrum of disease manifestations. Longitudinal, multicenter, multi-omic datasets integrated with digital phenotyping and network-level imaging must become the standard—not the exception. Only by moving beyond symptom palliation and toward mechanistically informed, stratified intervention can we hope to change the trajectory of PD.

## 8. Conclusions and Perspectives

Parkinson’s disease (PD) is a multisystem neurodegenerative disorder whose clinical expression extends far beyond its classical motor phenotype. The evidence synthesized throughout this review reinforces a transformative view of PD—one in which non-motor symptoms not only anticipate motor onset, but drive disease burden, accelerate disability, and profoundly affect the quality of life (see Figure 1 and Figure 2; Table 1, Table 2, Table 3, Table 4 and Table 5). These symptoms are neither incidental nor late-stage complications; they represent early and sustained disruptions in autonomic, cognitive, affective, and sensory networks. Despite this recognition, clinical frameworks and research priorities remain anchored in a dopamine-centric model that inadequately addresses the disease’s full complexity [2,3,4,5,6,27,129,131,229].

Non-motor symptoms—including REM sleep behavior disorder, constipation, hyposmia, depression, and cognitive dysfunction—often herald disease onset, yet they are frequently misdiagnosed or dismissed as age-related comorbidities. Their underrecognition impedes early diagnosis, limits timely intervention, and hinders therapeutic innovation. While the Braak staging hypothesis has provided a foundational framework for early pathology, inconsistencies in clinical correlation and α-synuclein topography across patient subtypes underscore the need to reassess this model in favor of a more integrative, systems-based approach [9,10].

The persistence of therapeutic stagnation—particularly in developing disease-modifying strategies—reflects a misalignment between pathophysiological insights and translational application. The neurocentric view of PD, centered on dopaminergic cell loss in the substantia nigra, has failed to account for parallel degeneration across serotonergic, cholinergic, noradrenergic, and glutamatergic systems. These circuits underlie many of the most debilitating non-motor symptoms, including cognitive impairment, mood disturbances, autonomic failure, and sleep dysfunction. Continued focus on dopamine alone cannot reverse or prevent the multisystem degeneration that characterizes PD.

Emerging technologies—such as multi-omic profiling, advanced neuroimaging, and digital biomarkers—offer the tools to redefine PD subtypes and identify at-risk individuals. However, these approaches require validation in large, longitudinal cohorts and integration into personalized clinical frameworks. Precision medicine in PD remains aspirational unless stratification efforts expand beyond motor phenotypes and incorporate non-motor domains from prodromal stages onward.

Cognitive impairment represents a defining, yet undertreated feature of PD. Mild executive deficits, often present at diagnosis, progress toward dementia in the majority of patients over time. The coexistence of α-synuclein pathology with Alzheimer’s-type tau and amyloid deposition in a subset of cases demands a broader conceptualization of PD-related cognitive decline. Current therapies, adapted from Alzheimer’s disease, yield only modest effects and ignore the dopaminergic and cholinergic interplay that shapes cognitive performance in PD. Redefining cognitive impairment as an early and mechanistically distinct manifestation of PD is essential to improve outcomes and delay dementia onset.

Similarly, the field has yet to address the clinical inertia surrounding sleep and autonomic dysfunction. Orthostatic hypotension, urinary symptoms, sialorrhea, constipation, and thermoregulatory instability remain poorly managed despite their high prevalence and early onset. These symptoms reflect central and peripheral autonomic failure, yet therapeutic strategies remain symptomatic, and their integration into routine care is limited. Sleep-related disorders, especially REM sleep behavior disorder, offer valuable diagnostic and prognostic insights, but are underutilized in practice and underrepresented in clinical trials.

Neuropsychiatric symptoms—including depression, anxiety, apathy, hallucinations, and impulse control disorders—emerge early, fluctuate with disease progression, and often coexist with cognitive decline. Their pathophysiology spans multiple neurotransmitter systems and neural circuits, yet pharmacological treatments remain extrapolated from non-neurological populations and rarely consider PD-specific mechanisms. Apathy in particular is frequently misdiagnosed and left untreated, despite its predictive value for cognitive deterioration. Addressing these symptoms requires dismantling artificial boundaries between neurology and psychiatry and adopting circuit-based models of behavioral dysfunction.

The gut–brain axis has opened promising avenues for rethinking PD origins. Enteric α-synuclein accumulation, altered microbiota composition, and increased intestinal permeability have been linked to early disease processes. However, the field has outpaced its evidence base, with many studies lacking mechanistic clarity or therapeutic validation. Intervening at the microbiome level remains an intriguing hypothesis, not a confirmed strategy. Future work must rigorously test whether peripheral interventions can alter central pathology.

The most pressing challenge in PD research is not a lack of hypotheses, but a reluctance to abandon outdated ones. The assumption that PD follows a singular pathological cascade, amenable to a universal intervention, has stymied therapeutic progress. The next generation of breakthroughs will arise not from refining dopaminergic therapies, but from reconceptualizing PD as a constellation of overlapping, subtype-specific syndromes. Embracing clinical and biological heterogeneity is not a limitation—it is the path forward.

This review does not seek to exhaustively catalogue every non-motor manifestation of PD. Instead, it offers a critical synthesis aimed at clinicians, researchers, and educators, advocating for reframing PD as a disorder of systems, not symptoms. To achieve meaningful advances, we must teach it as such, diagnose it accordingly, and treat it beyond its tremors. Non-motor symptoms are not ancillary—they are fundamental to the disease. The future of PD care depends on acknowledging this truth and building the diagnostic and therapeutic infrastructure to act on it.

## Figures and Tables

**Table 1:** Autonomic disorders in Parkinson’s disease.

Symptom	Prevalence and Stage	Biomarkers	Pharmacological Treatment	Non-Pharmacological Treatment
Sialorrhea	↑ 37–84%, early	↑ α-synuclein (basal ganglia)	Botulinum toxin, glycopyrrolate	Speech therapy, postural adjustments
Dysphagia	↑ 40–87%, early	↑ α-synuclein (enteric nervous system)	Botulinum toxin, levodopa	Swallowing therapy, neuromodulation
Constipation	↑ 40–63%, Prodromal	↑ α-synuclein (sacral nuclei)	Lubiprostone, prokinetics	Dietary fiber
Sexual dysfunction	↑ 65–90%, Early	No α-synuclein correlation	Sildenafil, hormone therapy	Psychotherapy, Couples therapy
Urinary dysfunction	↑ 25–61%, 5–6 years post-motor onset	↑ α-synuclein (pelvic plexus)	Antimuscarinics, beta-3 agonists	Bladder training, Pelvic floor exercises
Orthostatic hypotension	↑ 30–50%, early/late	↑ α-synuclein (autonomic nervous system)	Droxidopa, midodrine, fludrocortisone	↑ Salt/fluids, compression stockings
Seborrheic dermatitis	↑ 52–59%, early and progressive	↑ α-synuclein (sebaceous glands, dermal nerves)	Ketoconazole, cannabidiol	Skin care, microbiome modulation

↑ = Increased.

**Table 2:** Sensory disorders in Parkinson’s disease.

Symptom	Prevalence and Stage	Biomarkers	Pharmacological Treatment	Non-Pharmacological Treatment
Olfactory dysfunction	↑ 90%, prodromal	↑ α-synuclein (olfactory bulb, mucosa), ↓ functional activity	Intranasal insulin, DBS	Olfactory training
Ageusia	↑ 4–54%, mild-advanced	↓ Taste receptor gene expression, neurodegeneration	No specific treatment	Dietary adjustments
Visual disturbances	↑ 90%, prodromal	↑ α-synuclein (retina), ↓ dopamine, retinal atrophy	Dopaminergic therapy, artificial tears	Prism glasses, vision therapy
Pain	↑ 20–98%, prodromal	↑ α-synuclein (spinal cord, nerves), ↓ dopamine, ↑ neuroinflammation	NSAIDs, anticonvulsants, opioids	Physical therapy, CBT, DBS
Paresthesias	↑ 40%, prodromal	↑ α-synuclein (epidermal nerves), ↓ nerve fiber density	Dopaminergic meds, anticonvulsants	Sensory retraining

↑ = Increased; ↓ = Decreased; NSAID = nonsteroidal anti-inflammatory drug; DBS = deep brain stimulation; CBT = cognitive behavioral therapy.

**Table 3:** Sleep-related disorders in Parkinson’s disease.

Symptom	Prevalence and Stage	Biomarkers	Pharmacological Treatment	Non-Pharmacological Treatment
EDS	↑ 21–76%, early	↓ Hypocretin-1, ↑ Tau, ↑ α-synuclein	Modafinil, istradefylline, melatonin	Sleep hygiene, CBT, exercise
Insomnia	↑ 60–80%, early	↑ α-synuclein, associated with motor and cognitive symptoms	Melatonin, benzodiazepines	CBT-I, neuromodulation
OSA	↑ 45–66%, advanced	↑ Leptin, ghrelin, IL-6	Clonazepam, melatonin	CPAP therapy, sleep hygiene
RLS	↑ 20–40%, early	↓ Dopamine and serotonin, ↑ iron in brain	Dopaminergic agents, gabapentin	Exercise, thermotherapy
RBD	↑ 33–58%, preclinical	↑ Cognitive impairment risk, α-synuclein	Clonazepam, melatonin	Sleep safety modifications

↑ = Increased; ↓ = Decreased; EDS = excessive daytime sleepiness; OSA = obstructive sleep apnea; RLS = restless leg syndrome; RBD = REM sleep behavior disorder; CBT-I = cognitive behavioral therapy for insomnia; CPAP = continuous positive airway pressure.

**Table 4:** Neuropsychiatric disorders in Parkinson’s disease.

Symptom	Prevalence and Stage	Biomarkers	Pharmacological Treatment	Non-Pharmacological Treatment
Depression	↑ 35–45%, prodromal-late	↓ Thalamus and amygdala function	SSRIs, SNRIs, TCAs	CBT, TMS, ECT
Apathy	↑ 40–52%, early-late	↓ Mesocortical activity, VTA dysfunction	Dopamine agonists, rivastigmine	DBS, TMS
Anxiety	↑ 31%, prodromal-late	↑ TNF-α, ↓ nitric oxide	SSRIs, benzodiazepines	CBT, meditation
Visual hallucinations	↑ 27–50%, early-advanced	↓ Acetylcholine, cognitive decline	Rivastigmine, clozapine	CBT, music therapy
Phantosmia	↑ 0.5–18.2%, early-late	Correlation with hallucinations	Antiseizure, antipsychotics	Surgical intervention

↑ = Increased; ↓ = Decreased; VTA = ventral tegmental area; TNF-α = tumor necrosis factor alpha; SSRI = selective serotonin reuptake inhibitor; SNRI = serotonin–norepinephrine reuptake inhibitor; TCA = tricyclic antidepressant; CBT = cognitive behavioral therapy; TMS = transcranial magnetic stimulation; ECT = electroconvulsive therapy; DBS = deep brain stimulation.

**Table 5:** Cognitive disorders in Parkinson’s disease.

Symptom	Prevalence and Stage	Biomarkers	Pharmacological Treatment	Non-Pharmacological Treatment
Inattention and task-switching	↑ 20%, Preclinical	EEG alterations, gene mutations	Atomoxetine, methylphenidate	Cognitive training, physical activity
Bradyphrenia	↑ 25%, Early	↑ CSF metabolites, linked to constipation and bradykinesia	Levodopa, MAO-B inhibitors	Processing speed training
Dementia	↑ 80%, Late	↑ Cortical atrophy, ↑ α-synuclein, ↓ Amyloid-β	Donepezil, memantine, emerging therapies	Exercise, non-invasive brain stimulation
Impulse control disorders	↑ 20%, Early	↑ Dopamine tone, ↑ OFC metabolism	Adjust DRT, antipsychotics	CBT, DBS

↑ = Increased; ↓ = Decreased; EEG = electroencephalogram; CSF = cerebrospinal fluid; OFC = orbitofrontal cortex; MAO-B = monoamine oxidase B; DRT = dopamine replacement therapy; CBT = cognitive behavioral therapy; DBS = deep brain stimulation.

## Abbreviations

The following abbreviations are used in this manuscript:

α-syn	α-synuclein
CBT	cognitive behavioral therapy
CPAP	continuous positive airway pressure
CSF	cerebrospinal fluid
CSP	cortical silent period
DBS	deep brain stimulation
DMV	dorsal motor nucleus of the vagus
DRT	dopamine replacement therapy
EDS	excessive daytime sleepiness
EEG	electroencephalogram
FOp	frontal insular operculum
ICD	impulse control disorder
MAO-B	monoamine oxidase B
NBM	nucleus basalis of Meynert
NPDs	neuropsychiatric disorders
NSAID	nonsteroidal anti-inflammatory drug
OB	olfactory bulb
OFC	orbitofrontal cortex
OH	orthostatic hypotension
OSA	obstructive sleep apnea
PDD	Parkinson’s disease dementia
PFC	prefrontal cortex
RBD	REM sleep behavior disorder
REM	rapid eye movement
RLS	restless leg syndrome
SC	spinal cord
SD	seborrheic dermatitis
SN	substantia nigra
SNRI	serotonin–norepinephrine reuptake inhibitor
SSRI	selective serotonin reuptake inhibitor
TCA	tricyclic antidepressant
TMS	transcranial magnetic stimulation
TNF-α	tumor necrosis factor alpha
UPDRS	Unified Parkinson’s Disease Rating Scale
VTA	ventral tegmental area
